# Effect of roxadustat on lowering blood lipids in peritoneal dialysis patients with anemia ——**a multicenter, randomized, controlled clinical study**

**DOI:** 10.1080/0886022X.2025.2460726

**Published:** 2025-02-25

**Authors:** Tian Xu, Yilin Ruan, Na Liu, Yi Wang, Xiujuan Zang, Yuhua Ma, Hualin Qi, Xiuhua Mi, Geping Yu, Chunyan Zhang, Xiaomin Huang, Jingyuan Xie, Nan Chen, Hong Ren

**Affiliations:** a Department of Nephrology, Ruijin Hospital, Shanghai Jiao Tong University School of Medicine, Shanghai, China; b Department of Nephrology, Shanghai East Hospital, Tongji University School of Medicine, Shanghai, China; c Department of Nephrology, Shanghai Songjiang District Central Hospital, Shanghai, China; d Department of Nephrology, Traditional Chinese Medicine Hospital of Kunshan, Kunshan, Jiangsu, China; e Department of Nephrology, The People’s Hospital of Pudong New District in Shanghai, Shanghai, China; f Department of Nephrology, Shanghai Baoshan District Hospital of Integrated Traditional Chinese and Western Medicine, Shanghai, China; g Department of Nephrology, Tonglu First People’s Hospital, Tonglu, Hangzhou, China

**Keywords:** Roxadustat, blood lipids, peritoneal dialysis, anemia, iron metabolism

## Abstract

**Objectives:**

To observe the effect of roxadustat on lowering blood lipids in peritoneal dialysis (PD) patients beyond treating anemia.

**Methods:**

In a prospective, multicenter clinical study, we randomly assigned (in a 1:1 ratio) 100 PD patients who had received erythropoiesis-stimulating agent therapy for at least 4 weeks to receive either roxadustat or erythropoietin (EPO) for 48 weeks. The blood lipids, hemoglobin, blood pressure, blood glucose, iron metabolism and inflammatory factors were compared between the two groups at 0, 2, 4, 8, 12, 16, 20, 24 and 48 weeks, respectively.

**Results:**

At start of switching to roxadustat, hemoglobin seemed to rise a little faster (102.8 ± 15.4 vs. 97.1 ± 17.3 g/L at 2 weeks, *p* > 0.05), but there was no significant difference in hemoglobin change between the two groups over the course of observation (*p* = 0.185). At the early stage of the study (12 weeks), the transferrin saturation (TSAT) of roxadustat group decreased significantly from the baseline (32.7 (20.6) vs. 22.1 (18.7)%, *p* = 0.001). At the end of the study period (48 weeks), total cholesterol (3.89 ± 0.92 vs. 4.52 ± 1.14 mmol/L, *p* = 0.012), low density lipoprotein cholesterol (2.24 ± 0.74 vs. 2.63 ± 0.82 mmol/L, *p* = 0.045) and triglyceride (1.35 (0.86) vs. 1.89 (1.27) mmol/l, *p* = 0.013) in roxadustat group were significantly lower than those in EPO group.

**Conclusions:**

Roxadustat not only can improve anemia and iron metabolism, but also can reduce serum cholesterol and triglyceride levels in PD patients after switching from the EPO.

## Introduction

Dyslipidemia is common in chronic kidney disease (CKD) patients. The major causes of dyslipidemia in these population are nephrotic syndrome, decrease of glomerular filtration rate (GFR), use of immuno-suppressive agents, renal replacement therapy, comorbidity and nutritional status, etc. [1]. Elevated circulating lipid levels are one of several factors have been implicated in the increased cardiovascular risk associated with CKD [2]. Especially, cardiovascular disease (CVD) events among dialysis patients are 20- to 30-times higher than for the general population [3]. In the CKD patients, statin plus ezetimibe therapy led to a significant 17% reduction in the relative hazard of CVD events compared with placebo (HR 0.83; 95% CI 0.74–0.94) [4].

However, three major clinical research (SHARP, 4D, AURORA) [4–6] indicated that despite the exceedingly high cardiovascular risk in dialysis patients, statin regimens failed to lead to clinical benefit in dialysis population. Therefore, in the Kidney Disease Improving Global Outcomes (KDIGO) clinical practice guideline [7], initiation of statin treatment is not recommended for the dialysis patients. This may be due to the inability of statins to improve vascular calcification, cardiomyopathy, hyperkalemia and other CVD risk factors in this population. Unexpected, from the 48-month follow-up onwards, hyperlipidemia was independently associated with an increased risk of all-mortality in peritoneal dialysis (PD) patients, a recent study showed [8]. It may be necessary to provide a safe and reliable lipid-lowering drug other than statins to the PD patients.

Roxadustat is a first-in-class oral hypoxia-inducible factor (HIF) prolyl hydroxylase inhibitor (PHI) approved for anemia treatment in dialysis-dependent (DD) and Non-DD CKD [9,10]. The Phase III clinical trial not only confirmed the excellent effect of roxadustat in the treatment of anemia, but also found it significantly reduced total cholesterol by 17%, LDL by 24%, non-high-density lipoprotein (HDL) by 19%, and triglycerides by 8% in dialysis patients [9]. Roxadustat, meanwhile, did not cause liver damage which is common in the lipid-lowering drugs. In a word, the aim of this study was to observe the effect of roxadustat, a non-statin drug, on lowering blood lipids in PD patients beyond treating anemia.

## Methods

### Ethical approval

This prospective, multicenter, randomized, controlled clinical study was led by Shanghai Jiaotong University School of Medicine Ruijin Hospital. This study was approved by the Independent Scientific Advisory Committee of the Shanghai Jiaotong University School of Medicine Ruijin Hospital (Approval Number: 2020043). The study was conducted in accordance with the principles outlined in the Declaration of Helsinki. All enrolled patients had signed informed consent.

### Funding

Collaborative research project of Collaborative Innovation Center for Translational Medicine, Shanghai Jiao Tong University School of Medicine (TM201905), China Hospital Association, Blood Purification Center (CHABP2021-03)

### Participants

From 2020 to 2021, the patients from 7 hospitals in East China meeting the following inclusion criteria were enrolled. The study’s inclusion criteria were: (1) age more than 18 years, (2) end stage renal disease (ESRD) on adequate PD for a minimum of 3 months, (3) had received stable doses of erythropoietin (EPO) for more than 4 weeks, (4) the hemoglobin value during the screening period must have been 7.0–12.0 g/dL. The exclusion criteria were: (1) history of malignancy except for the following: cancers determined to be cured or in remission for more than 5 years, (2) chronic inflammatory disease other than glomerulonephritis that could have impacted erythropoiesis (e.g.,: rheumatoid arthritis, systemic lupus erythematosus), (3) life expectancy less than one year.

### Study design

**Figure 1.**
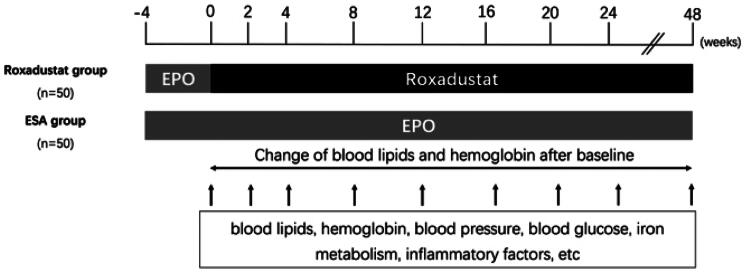
Study design.

100 Eligible patients underwent randomization in a 1:1 ratio to receive either oral roxadustat or erythropoietin (EPO, any brand) for 48 weeks. The study design is illustrated in Figure 1. The demographic and clinical data for these patients were obtained from their each visit. Fifty patients in the roxadustat group were switched from EPO injection to orally taking roxadustat three times per week and the other 50 patients in the control group continued EPO injection. The baseline for each participant was between June 2020 and November 2021.

The treatment protocol of EPO was based on the 2012 kidney disease improving global outcomes (KDIGO) clinical practice guideline for anemia in CKD [11]. The initial dose of roxadustat was either 70 mg (in patients weighing <60 kg) or 100 mg (in patients weighing >=60 kg). Doses were adjusted in order to maintain these patients’ hemoglobin levels within the range of 10.0–12.0g/dL. The patients who took statins before the study continued statin therapy, but the doses could not be increased or decreased during the follow-up. Statins could not be initiated in the other patients. All enrolled patients regularly received appropriate dietary guidance for ESRD. The use of oral iron therapy was allowed if necessary, intravenous iron therapy was prohibited except as rescue therapy. Rescue therapy meant intravenous iron in patients who had a hemoglobin level of less than 60 g/L. During the 48-week observation period, there were total 9 visits. Blood parameters (including hemoglobin, cholesterol, triglycerides, ferritin, transferrin saturation (TSAT)) were evaluated at each visit.

### Research objectives

Primary objectives were to compare the changes of the blood lipids and hemoglobin between the two groups. Secondary objectives were to compare the changes of blood pressure, blood glucose, iron metabolism, inflammatory factors between the two groups.

### Statistics

Data analysis was conducted using SPSS 22.0 statistical software (IBM Corp: Armonk, NY, USA, 2013). The quantitative variables were tested for normality by Kolmogorov–Smirnov method. Normally distributed quantitative variables were expressed as mean ± standard deviation, while non-normally distributed quantitative variables were expressed as median and interquartile range (IQR). Comparison between groups was performed by *t* test for normal distribution variables or Wilcoxon rank sum test for non-normal distribution variables. Two-tailed significance was defined as *p* < 0.05.

At first, Mauchly’s Tests were conducted to assess whether the assumption of sphericity was met. After that, hemoglobin, blood pressure, fasting blood glucose, glycated hemoglobin, and serum iron, which were normally distributed quantitative variables were selected. These variables were further compared over time by repeated measures Analysis of Variance (ANOVA). Information of lost follow-up or death cases during the observation period was regarded as censored data.

## Results

### Patient demographics and baseline characteristics

**Figure 2.**
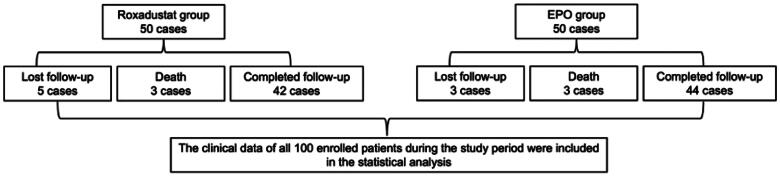
Outcomes of enrolled patients.

As of November 2022, all patients’ follow-up had been completed. A total of 5 patients in the roxadustat group lost follow-up (2 cases received kidney transplantation, 1 case transferred to hemodialysis, 1 case was admitted to a rehabilitation hospital, and 1 case returned to hometown), while 3 cases in the EPO group lost follow-up (1 case received kidney transplantation, 1 case transferred to hemodialysis, 1 case returned to hometown). There were a total of 6 deaths, including 3 cases in the roxadustat group (1 case of peritonitis, 1 case of cerebral infarction, and 1 case of arrhythmia) and the other 3 cases in the EPO group (1 case of peritonitis, 1 case of gastrointestinal hemorrhage, and 1 case of heart failure). A total of 86 PD patients, including 42 cases in the roxadustat group and 44 cases in the EPO group, had completed 48 weeks’ follow-up. The clinical data of all 100 enrolled patients during the study period were included in the statistical analysis. (Figure 2).

At baseline, the patient demographics, blood pressure, dialysis adequacy, hemoglobin, glucose metabolism, electrolytes, iron metabolism, inflammatory factors, and lipid levels were similar between the two groups (Table 1).

**Table 1.** Patient demographics and laboratory exam at baseline.

	Roxadustat (*n* = 50)	EPO (*n* = 50)	*p* Value
Gender (male: female)	28: 22	29: 21	1.00
Age (years)	51.56 ± 13.15	55.06 ± 13.46	0.17
Dialysis duration (months)	26.5 (55.3)	24.6 (49.6)	0.34
BMI (Kg/m^2^)	23.70 ± 4.26	24.06 ± 3.13	0.55
SBP (mmHg)	142.64 ± 21.15	144.63 ± 21.11	0.84
DBP (mmHg)	84.31 ± 11.78	84.59 ± 15.12	0.50
Total KT/Vurea	2.25 ± 0.61	2.16 ± 0.54	0.22
Total Ccr (l/W)	59.56 (26.97)	52.76(27.52)	0.08
Hemoglobin (g/L)	94.93 ± 17.78	98.44 ± 15.40	0.71
FBG (mmol/L)	5.94 ± 1.30	5.39 ± 1.30	0.52
Glycosylated Hb (%)	5.80 ± 1.24	5.82 ± 0.93	0.97
Albumin (g/L)	33.37 ± 4.63	34.97 ± 5.04	0.61
Creatinine (umol/L)	881(420)	877(515)	0.46
UA (umol/L)	383(130)	402(86)	0.09
Calcium (umol/L)	2.19(0.30)	2.28(0.28)	0.43
Phosphorus (umol/L)	1.75(0.62)	1.57(0.76)	0.68
Iron (umol/L)	15.19 ± 7.77	15.71 ± 6.25	0.61
Ferritin (ng/mL)	160.4(282.15)	191.4(208.8)	0.21
TSAT (%)	32.7(20.6)	38.1(20.3)	0.07
hs-CRP (mg/L)	1.87(4.5)	2.67(6.61)	0.08
ESR (mm/min)	44(29.5)	44(41.5)	0.53
TG (mmol/L)	1.77(1.13)	2.06(1.33)	0.07
TC (mmol/L)	4.30 ± 1.26	4.66 ± 1.37	0.09
LDL (mmol/L)	2.65 ± 1.00	2.87 ± 1.09	0.41
HDL (mmol/L)	1.00 ± 0.33	1.03 ± 0.38	0.81

BMI: Body mass index; SBP: Systolic blood pressure; DBP: Diastolic blood pressure; KT/Vurea: Urea clearance index; Ccr: Creatinine clearance rate; FBG: Fasting blood glucose; Glycosylated Hb: Glycosylated hemoglobin; UA: Uric acid; TSAT: Transferrin saturation; hs-CRP: Hypersensitive C-reactive protein; ESR: Erythrocyte sedimentation rate; TG: Triglyceride; TC: Total cholesterol; LDL: Low density lipoprotein; HDL: High density lipoprotein.

### Effect of roxadustat on anemia

**Figure 3.**
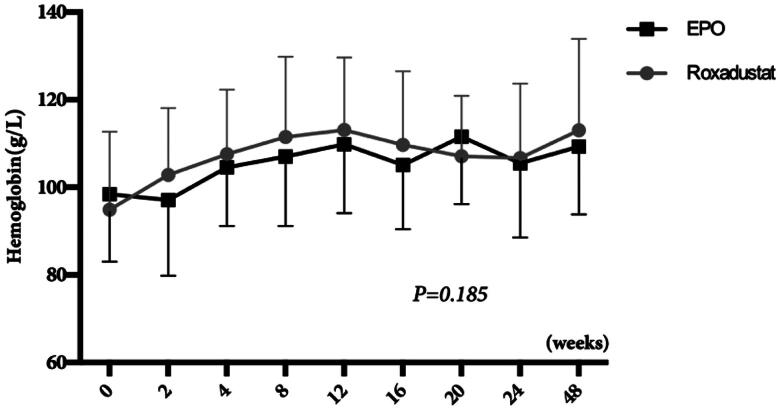
Changes in hemoglobin.

The hemoglobin concentration significantly increased from 94.9 ± 17.8 g/L at baseline to 102.8 ± 15.3 g/L at 2 weeks (*p* < 0.05), 107.6 ± 14.7 g/L at 4 weeks (*p* < 0.01), 111.5 ± 18.3 g/L at 8 weeks (*p* < 0.01), 113.1 ± 16.5 g/L at 12 weeks (*p* < 0.01), 109.7 ± 16.8 g/L at 16 weeks (*p* < 0.01), 107.1 ± 13.8 g/L at 20 weeks (*p* < 0.01), 106.7 ± 17.0 g/L at 24 weeks (*p* < 0.01), 113.1 ± 20.9 g/L at 48 weeks (*p* < 0.01) in the roxadustat group, whereas the EPO group’s hemoglobin concentrations did not change significantly at the early stage of the study (98.4 ± 15.4 g/L at baseline vs. 97.1 ± 17.3 g/L at 2 weeks, *p* > 0.05). As shown in Figure 3, at start of switching to roxadustat, hemoglobin seemed to rise a little faster (102.8 ± 15.4 vs. 97.1 ± 17.3 g/L at 2 weeks, *p* > 0.05), but there was no significant difference in hemoglobin change between the two groups over the course of observation (*p* = 0.185).

### Effect of roxadustat on blood pressure and glucose metabolism

**Figure 4.**
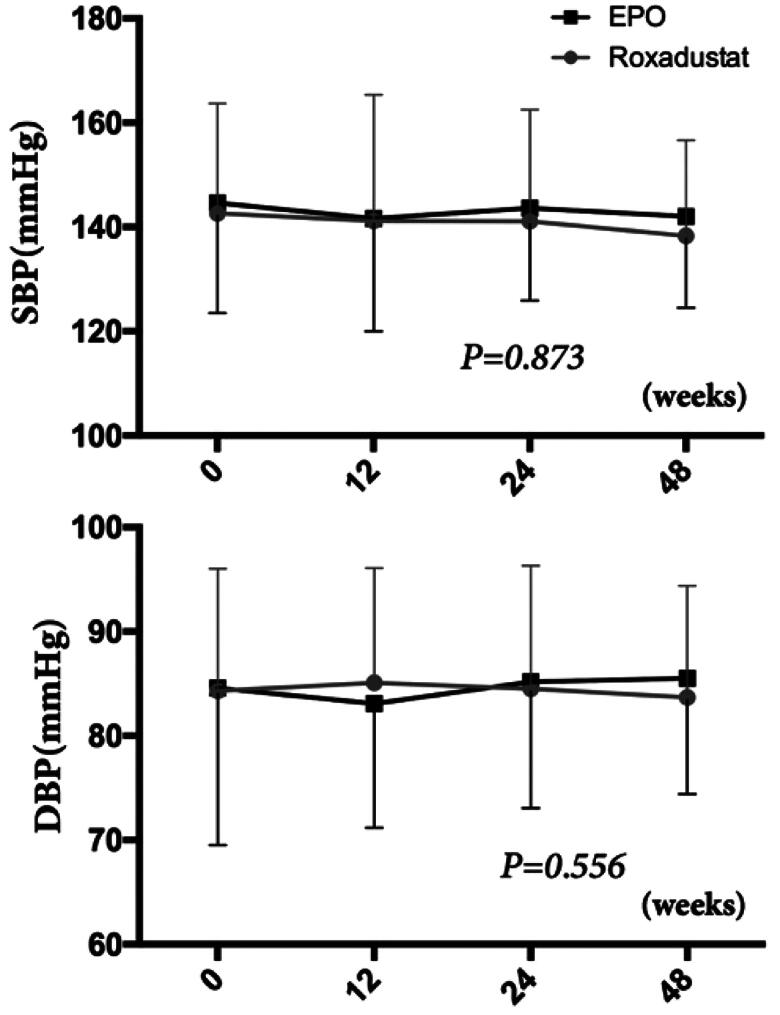
Changes in blood pressure. SBP: Systolic blood pressure; DBP: Diastolic blood pressure.

**Figure 5.**
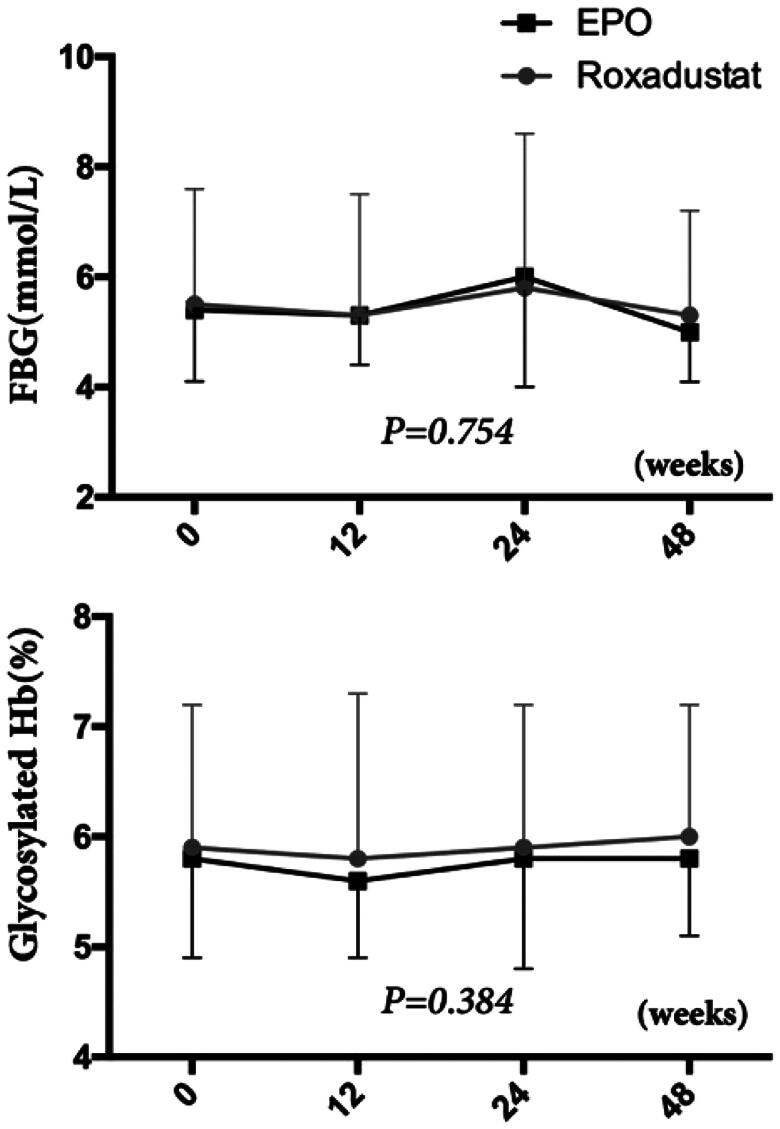
Changes in glucose metabolism. FBG: Fasting blood glucose; Glycosylated Hb: Glycosylated hemoglobin

At baseline, there were no significant differences in systolic blood pressure (SBP) (142.6 ± 21.1 vs. 144.6 ± 21.1 mmHg, *p* > 0.05) and diastolic blood pressure (DBP) (84.3 ± 11.7 vs. 84.5 ± 15.1 mmHg, *p* > 0.05) between the two groups. Also, both of SBP (*p* = 0.873) and DBP (*p* = 0.556) did not significantly change over the study term in either group (Figure 4). At week 0, the fasting blood glucose (FBG, 5.5 ± 2.1 vs. 5.4 ± 1.3 mmol/L, *p* > 0.05) and glycosylated hemoglobin (Hb, 5.9 ± 1.3 vs. 5.8 ± 0.9%, *p* > 0.05) levels were similar between the two groups. Moreover, there were no change in FBG and glycosylated Hb between before and after baseline in either the roxadustat or EPO groups (Figure 5).

### Changes in inflammatory factors

**Figure 6.**
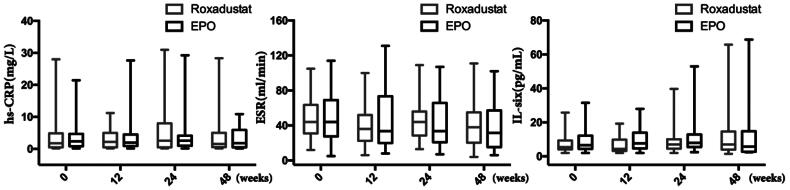
Changes in inflammatory factors. hs-CRP: Hypersensitive C-reactive protein; ESR: Erythrocyte sedimentation rate; IL-six: Interleukin-six.

The inflammatory factors, including hypersensitive C-reactive protein (hs-CRP), erythrocyte sedimentation rate (ESR), interleukin-6 (IL-6) did not significantly differ from their baseline values in either the roxadustat or EPO groups at 12, 24 and 48 weeks. There were also no significant differences in the inflammatory factors between the two groups during whole term of this study (Figure 6).

### Effects of roxadustat on iron metabolism

**Figure 7.**
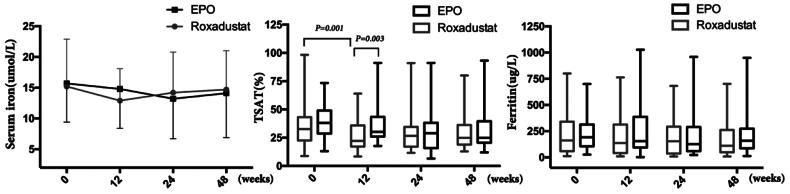
Changes in iron metabolism. TSAT: Transferrin saturation.

Both of serum iron and ferritin concentration did not signi­ficantly change during the study in either group. However, the TSAT decreased obviously from 32.7 (20.6)% at 0 week to 22.1 (18.7)% at 12 weeks (*p* = 0.001) in the roxadustat group, and TSAT concentration in the roxadustat group was significantly lower than that in the EPO group (22.1 (20.6) vs. 30.1 (17.3)%, *p* = 0.003) at 12 weeks (Figure 7).

### Effect of roxadustat on lowering total cholesterol

**Figure 8.**
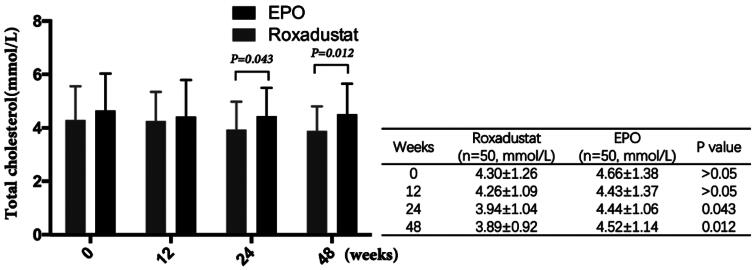
Changes in total cholesterol.

There was no significant difference in total cholesterol (TC) between the two groups at baseline (4.30 ± 1.26 vs. 4.66 ± 1.38 mmol/L, *p* > 0.05), but TC in the roxadustat group was significantly lower than that in the EPO group at 24 weeks (3.94 ± 1.04 vs. 4.44 ± 1.06 mmol/L, *p* = 0.043). By the end of the study, the decrease of total cholesterol in the roxadustat group was more markedly (3.89 ± 0.92 vs. 4.52 ± 1.14 mmol/L, *p* = 0.012) (Figure 8).

### Effect of roxadustat on lowering low density lipoprotein

**Figure 9.**
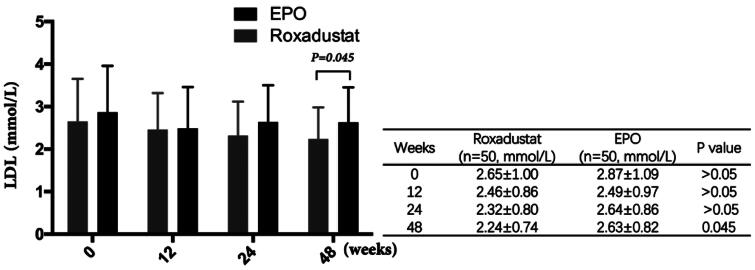
Changes in LDL. LDL: Low density lipoprotein.

At 0, 12 and 24 weeks, there were no significant difference in low density lipoprotein (LDL) between the two groups (*p* > 0.05). However, LDL was significantly lower in roxadustat group than that in the EPO group at 48 weeks (2.24 ± 0.74 vs. 2.63 ± 0.82 mmol/L, *p* = 0.045) (Figure 9).

### Effect of roxadustat on lowering triglyceride

**Figure 10.**
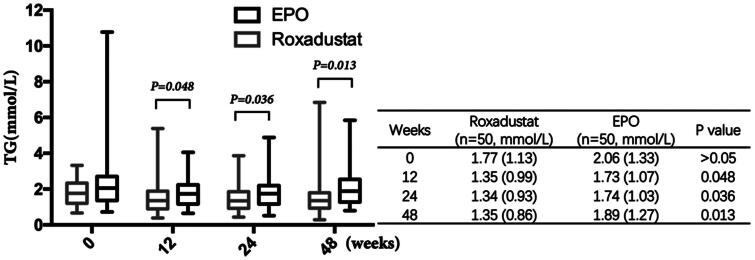
Changes in TG. TG: Triglyceride.

Although there was no significant difference in triglyceride between the two groups at week 0 (1.77 (1.13) vs. 2.06 (1.33) mmol/L, *p* > 0.05), triglyceride in roxadustat group was significantly lower than that in EPO group from week 12 (1.35 (0.99) vs. 1.73 (1.07)mmol/L, *p* = 0.048). Moreover, the difference in triglyceride between the two groups became more significant from then on. (week 24: 1.34 (0.93) vs. 1.74 (1.03), *p* = 0.036; week 48: 1.35 (0.86) vs. 1.89 (1.27) mmol/l, *p* = 0.013) (Figure 10).

## Discussion

Roxadustat is an inhibitor of HIF prolyl hydroxylase (HIF-PHI), which stabilizes HIF and stimulates the expression of erythropoietin gene. This newly marketed drug increases the concentration of erythropoietin in the kidney and liver to the physiological range so as to increase or maintain the Hb concentration in CKD patients with anemia [12]. Although the two randomized, multicenter, large-scale, phase 3, clinical trial had already revealed that roxadustat maintained the Hb concentrations both in dialysis-dependent (DD) and non-dialysis dependent (NDD) patients [9,10], PD patients accounted for only a small proportion of the enrolled populations (33/304, 10.8%). In this study that only limited to PD patients, the increase rate of Hb concentration in roxadustat group was significantly accelerated after switching from EPO therapy. From week 8, with the doses of roxadustat were gradually tapered and maintained, the Hb concentrations were similar between the two groups. Consistent with the result of the phase 3 clinical trial, dose of EPO required to maintain the target Hb concentration increased, while the dose of roxadustat that was required decreased [9]. The result indicates that Roxadustat can still stabilize Hb concentrations when the doses were gradually tapered in PD patients with anemia who previously were treated with EPO.

As a vasoconstrictor, EPO not only can raise blood pressure directly, but also can stimulate the production of endothelin, epinephrine, renin, and angiotensin which elevate the blood pressure. Moreover, the effect on blood viscosity is also a mechanism of hypertension in EPO [13]. Therefore, ESRD patients usually have higher blood pressure after EPO applied. A retrospective study that only limited to PD patients showed PD patients treated with roxadustat had lower blood pressure and better cardiovascular parameters than those treated with EPO [14]. Same as the results of another only PD patients limited study [15], blood pressure did not significantly differ from their baseline values in either the Roxadustat or ESA groups at 12, 24, 48 weeks in our study.

In an animal model of nephropathy with metabolic syndrome, HIF-PHI can regulated glucose metabolism and reduced fat weight by increasing renal glucose excretion [16]. Inhibition of prolyl hydroxylase (PHD) can also improve insulin resistance and lower serum insulin levels. Yet, our results showed roxadustat did not significant change the level of fasting blood glucose and glycosylated Hb. In the patients new to PD [17], CRP levels in the roxadustat group were significantly lower than those in the EPO group, which may be related to Roxadustat promoting adenosine gene expression and assisting adenosine to exert anti-inflammatory effects. The potential anti-inflammatory effect of Roxadustat had been identified in an earlier animal experiment [18]. In the present study, the results of hs-CRP, ESR and IL-6 indicated that roxadustat didn’t improve the inflammatory status of PD patients.

Roxadustat has been identified to improve iron metabolism in a variety of ways. It increases the absorption of iron by the gastrointestinal tract, promotes the release of stored iron from hepatocytes, and enhances the total serum iron-binding capacity [12]. A previous clinical study showed that administration of roxadustat reduced serum ferritin and TSAT in PD patients after switching from EPO [19]. In our study, the roxadustat group’s TSAT concentration decreased significantly from baseline at 12 weeks, and the roxadustat group’s TSAT concentration was significantly lower than that in the EPO group at 12 weeks. This suggested that roxadustat had significantly promoted iron utilization at an early stage.

The roles of roxadustat in correcting anemia and improving iron metabolism have been consistently acknowledged. The main aim of our study was to observe the effect of Roxadustat on lowering blood lipids in PD patients in addition to correcting anemia. In the Phase III clinical trial, it had already been found that roxadustat could significantly reduce total cholesterol, LDL and triglycerides in dialysis patients [9]. While, above-mentioned study only enrolled 33 PD patients, who accounted for only 10.8% of all participants. We found that the concentrations of TC and LDL in roxadustat group decreased dramatically at middle and late stages of our study. Meanwhile, triglyceride (TG) concentration in roxadustat group decrease even earlier.

There are several reasons for the apparent failure of statins in reducing CVD events and mortality in the dialysis patients [20]. First, the pathophysiology and spectrum of CVD in dialysis patients are markedly different compared to that in the general population. Second, except atherosclerosis, dialysis patients are also exposed to anemia, inflammation, oxidative stress, etc. Third, the presence of malnutrition and inflammation in ESRD patients may alter the relationship between cholesterol and CVD. Fourth, highly atherogenic lipoproteins and their fragments such as very low density lipoprotein (VLDL), small dense-LDL and lipoprotein (a) accumulate prominently in dialysis patients. In other words, a novel drug that can simultaneously treat anemia, improve inflammatory states, and lower VLDL and l lipoprotein (a) may help to reduce CVD events in dialysis patients.

The main underlying mechanism of roxadustat involved in lowering cholesterol levels perhaps is that HIF-1 accelerates degradation of HMG-CoA reductase in the liver through activation of insulin-induced gene 2 transcription, leading to reduced cholesterol synthesis [21]. Another explanation is that HIF increases the expression of the target gene ATP- binding cassette transporter A1, which mediates the overflow of intracellular cholesterol and phospholipid to apolipoprotein deficiency, resulting in a decrease in intracellular cholesterol [22]. Moreover, HIF-1 also stimulates lipin 1 gene expression, which contributes to TG accumulation in cells [23]. By these mechanisms, roxadustat stabilizes HIF and then reduce TC, LDL-C, and TG levels. While, so far, it is still unknown whether roxadustat could affect VLDL or lipoprotein (a) levels.

Except for the phase III clinical trial from Japan [19], all of the previous studies on roxadustat which were limited to PD patients were either single-center, retrospective, single-arm, or small sample size. After roxadustat been marketed, the present attempt is only limited to PD patients, prospective, randomized control, multicenter clinical study in order to explore the effects of treating anemia and lowering blood lipids in PD patients. However, the present study also had several limitations. First, we did not record the CVD events during follow-up, therefore the incidence of CVD events can’t be compared between the two groups. Second, the VLDL and lipoprotein (a) levels weren’t tested in our study, so the effect of roxadustat on these lipid parameters can’t be evaluated, as well. Third, we did not collect the possible side effects of this medication, such as the association between the dose of roxadustat and serum potassium concentration. Due to the relatively small amount of participants in this study and short follow-up time, we didn’t observe any difference in cumulative and CVD survivals between the two groups.

In conclusion, roxadustat not only can improve anemia and iron metabolism, but also can reduce serum cholesterol and triglyceride levels in PD patients after switching from the EPO therapy. Whether roxadustat can reduce the incidence of CVD events and improve the survival in PD patients require the studies with larger sample sizes and longer follow-up time to further explore.
